# Rapid Chromosome Evolution in Recently Formed Polyploids in *Tragopogon* (Asteraceae)

**DOI:** 10.1371/journal.pone.0003353

**Published:** 2008-10-09

**Authors:** K. Yoong Lim, Douglas E. Soltis, Pamela S. Soltis, Jennifer Tate, Roman Matyasek, Hana Srubarova, Ales Kovarik, J. Chris Pires, Zhiyong Xiong, Andrew R. Leitch

**Affiliations:** 1 School of Biological and Chemical Sciences, Queen Mary College, University of London, London, United Kingdom; 2 Department of Botany and the Genetics Institute, University of Florida, Gainesville, Florida, United States of America; 3 Florida Museum of Natural History and the Genetics Institute, University of Florida, Gainesville, Florida, United States of America; 4 Massey University, Institute of Molecular Biosciences, Palmerston North, New Zealand; 5 Institute of Biophysics, Academy of Sciences of the Czech Republic, Brno, Czech Republic; 6 Division of Biological Sciences, Life Sciences Center, University of Missouri, Columbia, Missouri, United States of America; University of Massachusetts Amherst, United States of America

## Abstract

**Background:**

Polyploidy, frequently termed “whole genome duplication”, is a major force in the evolution of many eukaryotes. Indeed, most angiosperm species have undergone at least one round of polyploidy in their evolutionary history. Despite enormous progress in our understanding of many aspects of polyploidy, we essentially have no information about the role of chromosome divergence in the establishment of young polyploid populations. Here we investigate synthetic lines and natural populations of two recently and recurrently formed allotetraploids *Tragopogon mirus* and *T. miscellus* (formed within the past 80 years) to assess the role of aberrant meiosis in generating chromosomal/genomic diversity. That diversity is likely important in the formation, establishment and survival of polyploid populations and species.

**Methodology/Principal Findings:**

Applications of fluorescence *in situ* hybridisation (FISH) to natural populations of *T. mirus* and *T. miscellus* suggest that chromosomal rearrangements and other chromosomal changes are common in both allotetraploids. We detected extensive chromosomal polymorphism between individuals and populations, including (i) plants monosomic and trisomic for particular chromosomes (perhaps indicating compensatory trisomy), (ii) intergenomic translocations and (iii) variable sizes and expression patterns of individual ribosomal DNA (rDNA) loci. We even observed karyotypic variation among sibling plants. Significantly, translocations, chromosome loss, and meiotic irregularities, including quadrivalent formation, were observed in synthetic (S_0_ and S_1_ generations) polyploid lines. Our results not only provide a mechanism for chromosomal variation in natural populations, but also indicate that chromosomal changes occur rapidly following polyploidisation.

**Conclusions/Significance:**

These data shed new light on previous analyses of genome and transcriptome structures in *de novo* and establishing polyploid species. Crucially our results highlight the necessity of studying karyotypes in young (<150 years old) polyploid species and synthetic polyploids that resemble natural species. The data also provide insight into the mechanisms that perturb inheritance patterns of genetic markers in synthetic polyploids and populations of young natural polyploid species.

## Introduction

Polyploidy has played a major role in generating angiosperm biodiversity. Chromosome counts suggest that between 30 and 80% of angiosperm species are polyploid, while genomic studies of selected model and crop species reveal evidence of extensive ancient genome-wide multiplications. Indeed recent genomic investigations indicate that most, if not all, angiosperm species have undergone at least one genome duplication event in their evolutionary history, and several have evidence of multiple polyploidy-diploidisation-polyploidy cycles [1], [2], [3].

Angiosperm genomes are astonishingly plastic in their ability to tolerate considerable karyotypic (e.g. chromosome number variation, translocations), genetic (mutations, retroelement transpositon, deletions) and epigenetic (DNA methylation, histone methylation/acetylation) variability. This tolerance enables polyploids to form and establish and has contributed significantly to their widespread occurrence [4]. Large-scale genetic changes induced by polyploidy are thought to influence the transcriptome, metabolome and proteome, which can concomitantly alter the phenotype and ecology of the individuals. Most of the new genetic changes are probably maladaptive, but in a few rare instances individuals arise that are able to outcompete the parental diploids or colonize new niches.

There are several examples of recent speciation via polyploidy that occurred within the last 150 years: *Spartina anglica* [5], *Senecio cambrensis* and *S. eboracensis* [6], *Cardamine schulzii* [7], *Tragopogon mirus* and *T. miscellus* [8]. Studies on the genetic consequences of allopolyploidy in these *de novo* polyploid species reveal some significant differences. In *S. anglica* allopolyploidy induced few changes in genome structure, but there is epigenetic reprogramming [5], [9], while in *Senecio* [10], [11] and *Tragopogon* [12] allopolyploids there are substantial genetic changes including loss of sequence (genomic DNA profiles) and perturbations to the transcriptome (cDNA profiles). However, little attention has been paid to the chromosomal content of any of these allopolyploids, and it is unknown what mechanisms drive the observed genetic changes. For example, genetic change could be driven by local mutation, small-scale deletion or insertion of a particular sequence, or via major chromosomal changes, including whole-arm transposition and chromosome losses or duplications. Because there is little or no understanding of chromosomal variation in these recently formed, natural polyploids, we have embarked on a characterization of karyotypic variation between individuals and populations of the two *Tragopogon* allopolyploids from North America.

**Figure 1:**
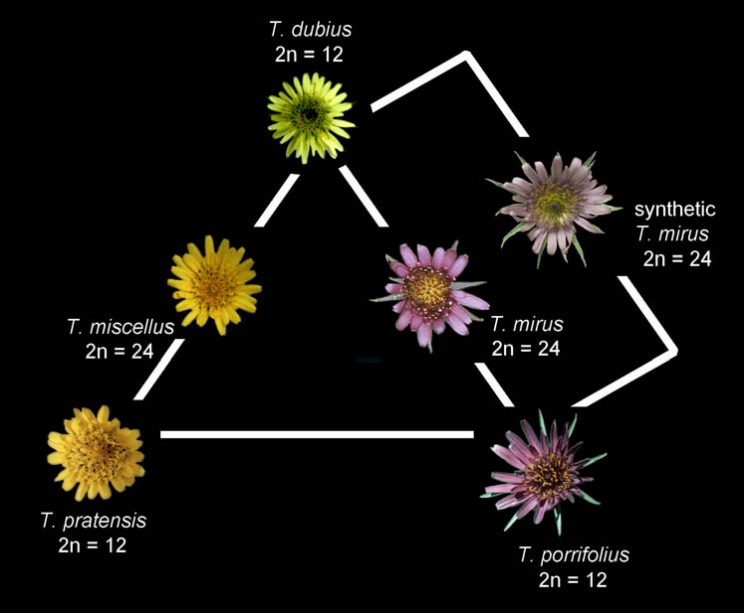
Tragopogon triangle with the flowers of the diploid *Tragopogon* species at the apices of the triangle. The flowers of the polyploids *T. miscellus* and *T. mirus* are shown between their respective diploid parents. The synthetic polyploid described here has the same parents as *T. mirus*.

*Tragopogon mirus* and *T. miscellus* have proved to be excellent evolutionary model systems for understanding early allopolyploid formation. These allopolyploids are derived from three diploid progenitors (each 2*n* = 2*x* = 12), *T*. *pratensis*, *T*. *porrifolius* and *T. dubius*, the latter being shared by both allopolyploids (*T. mirus* derived from *T*. *dubius*×*T*. *porrifolius*, 2*n* = 4*x* = 24; *T. miscellus* derived from *T*. *dubius*×*T*. *pratensis*, 2*n* = 4*x* = 24, Figure 1) [13]. The diploid parents of both polyploids are in well separated clades [14] and are not closely related based on ITS/ETS sequences, allozymes and other genetic markers [15], [16]. There are many reports of natural F_1_ hybrids involving these diploids and we have also produced them in the glasshouse, but these hybrids are highly sterile suggesting minimal homeologue pairing at meiosis [8]. In contrast the allopolyploids *T. mirus* and *T. miscellus* are fertile and expanded their ranges rapidly after their initial formations, in large part via multiple origins [17]. The two allotetraploids now occupy a large geographic area of eastern Washington and adjacent Idaho, USA, and comprise many thousands of individuals in several populations. Molecular analyses have revealed that *T. mirus* has recurrently formed at least 13 times and *T. miscellus* possibly as many as 21 times [13,16,18,19,20, Symonds et al. unpubl.,21], reviewed in Soltis *et al.* [8]. Furthermore, there are genetic differences between populations of each tetraploid, most of which likely reflect variation found in the diploids and inherited in the polyploid populations through recurrent formation, and others of which may have arisen through subsequent divergence of the polyploids. Examples of the latter include the divergence of rRNA gene copy number, sequence homogeneity and expression patterns [22], [23]. Here we show that population differences are also reflected in substantial variability in karyotypes among individuals, differences that appear correlated with irregular meiosis.

## Results

### Genome structure of *Tragopogon* allotetraploids

**Figure 2:**
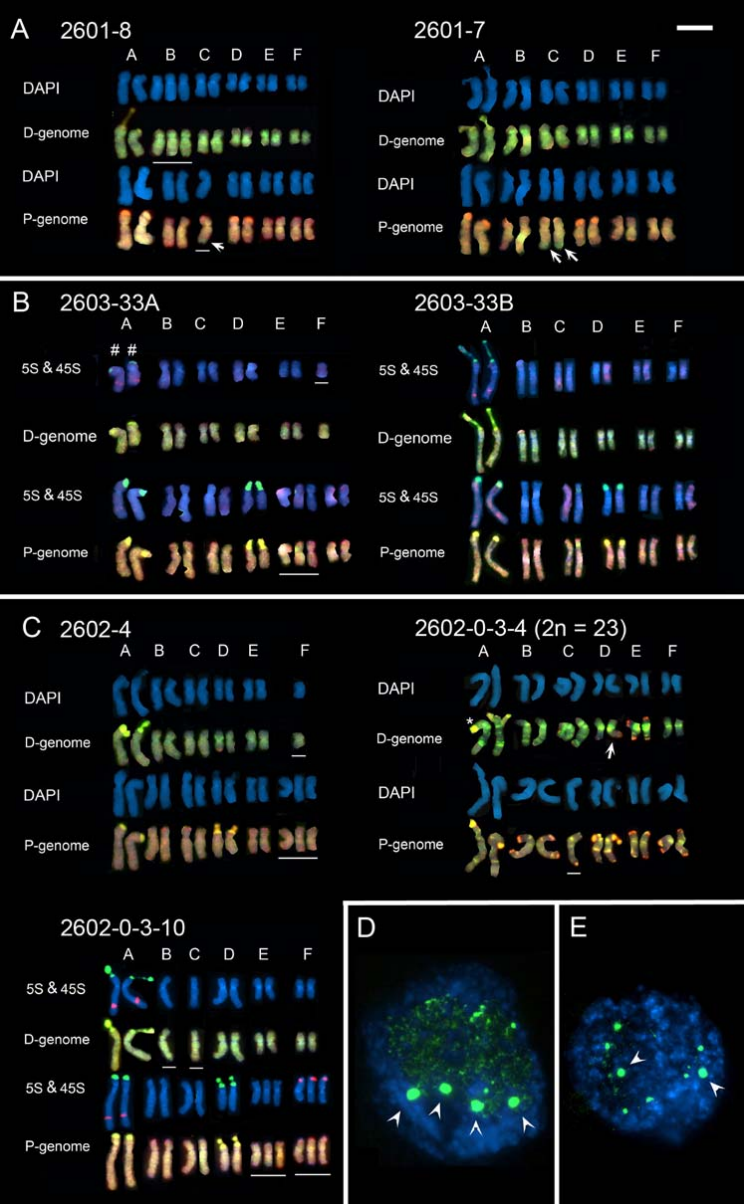
(A-C) Karyotype analyses of *T. mirus* from individuals in three populations (A) 2601, (B) 2603 and (C) 2602. Homeologous chromosome group nomenclature (A-F) follows Ownbey and McCollum [25] and Pires *et al.* [24]. Fluorochrome colours: yellow/green (FITC, digoxigenin-labelled probes), orange/red (Cy3, biotin-labelled probes), blue (DAPI staining). Each karyotype is shown with DAPI staining, sometimes also simultaneously labelled for 45S rDNA (yellow fluorescence) or 5S rDNA (red fluorescence), and after GISH with *T. dubius* (green fluorescence) and *T. porrifolius* (red fluorescence) total genomic DNA probes. Monosomic or trisomic chromosomes are underlined. Note: (1) intergenomic translocations to chromosome C^po/du^ (arrows) in individuals 2601-7 and 2601-8 and D^du/po^ in individual 2602-0-3-4 (A). (2) The 45S rDNA locus on the two homologues of chromosome A^du^ in individuals 2602-4 and 2602-0-3-4 show different levels of decondensation, one being condensed and the other with a secondary constriction (C). (3) A large reduction in size of the 45S rDNA locus on chromosome A^du^ of individual 2603-33A (#) compared with other individuals (D). (4) No secondary constrictions of 45S rDNA loci on *T. porrifolius* origin chromosomes (A^po^, D^po^) in individual 2603-33B. (D-E) Root-tip interphase nuclei of *T. mirus* after FISH with digoxigenin-labelled pTa71 for 45S rDNA. (D) Individual 2601-4, showing four large condensed 45S rDNA loci (arrows) and (E) Individual 2602-0-3-10 with two large condensed 45S rDNA loci (arrows). Scale bar (top right) is 10 µm.

**Figure 3 pone-0003353-g003:**
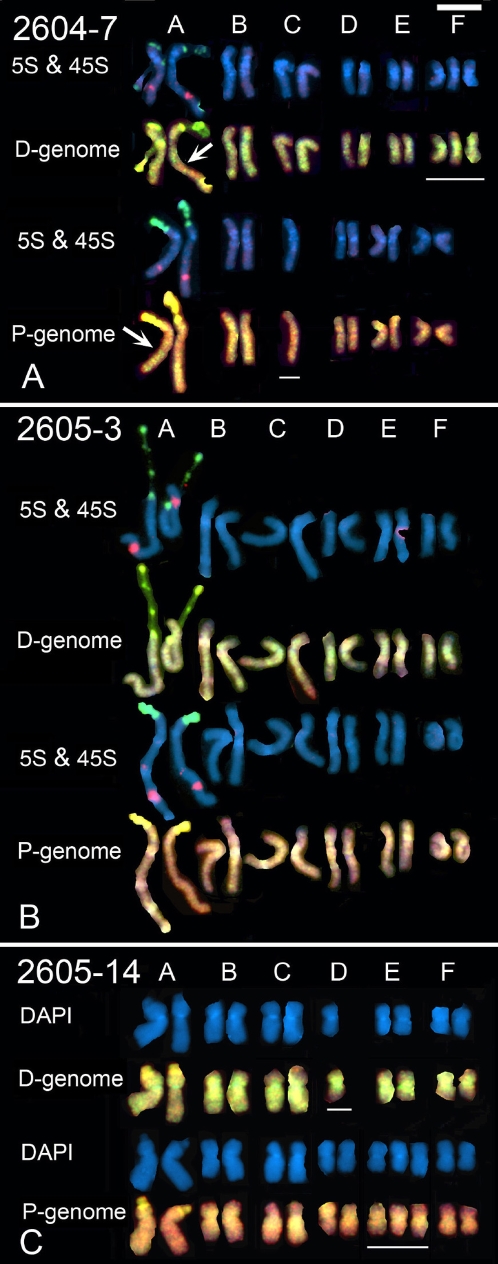
(A–C) Karyotype analyses of *T. miscellus* from two populations (A) 2604 and (B) 2605. Homeologous chromosome group nomenclature (A–F) follows Ownbey and McCollum [25] and Pires *et al*. [24]. Fluorochrome colours and probes are as in Figure 2 except that biotinylated *T. pratensis* genomic DNA was used in GISH experiments. Monosomic and trisomic chromosomes are underlined. Note in (A) that there is a large intergenomic translocation, chromosome A^du/pr^ (arrow). Scale bar (top right) is 10 µm.

The chromosome sizes, numbers and centromere indices are similar between the parental diploid species, and three tandem repeats characterized by us do not generate differences in chromosomal distribution. For these reasons we are unable to identify the parental origin of the chromosomes in the derived allotetraploid species via morphology alone [24]. We therefore used FISH with total genomic DNA probes (called Genomic In Situ Hybridization or GISH) to determine the genomic composition of karyotypes of *T. mirus* and *T. miscellus* individuals (Figures 2 and 3). We analysed 12 plants, nine of *T. mirus* from three populations (Table 1) and three of *T. miscellus*, two from one population and a third individual from a second population (Table 2). Five of these plants, including representatives of each tetraploid, were chosen because we had previously observed [22], [23] that they had particular 45S nuclear ribosomal DNA (45S rDNA) compositions and expression characteristics (Table 3). GISH labelling enabled the genomic origin of the chromosomes to be determined by fluorescence colour: digoxingenin-labelled genomic DNA of *T. dubius* labelled chromosomes of *T. dubius* origin green or yellow (D-genome) and biotin-labelled genomic DNA of either *T. pratensis* (to *T. miscellus*) or *T. porrifolius* (to *T. mirus*) labelled the P-genome (either *T. pratensis* or *T. porrifolius*) orange or red. However, the distinction between genomes was only possible after electronic merger of the images because there was considerable cross-hybridisation of probes. The homologous group assigned to each chromosome (A–F) was determined using size, arm ratio, position of 5S and 45S rDNA, as described in Pires et al. [24], and by using DAPI bright bands that were revealed after denaturation in some metaphases.

**Table 1:** Karyotype organisation of individuals of *T. mirus* from three populations (localities of each population are indicated in column 1).

*T. mirus* population	Plant number	Chromosome No. (2n)	Nature of karyotype imbalance		Translocations
			*T. dubius* origin	*T. porrifolius* origin	
2601 Pullman, Washington	4	24	E	E	–
	7	24	E	E	2×C^po/du^
	8	24	3×B^du^	1×C^po^	1×C^po/du^
2602 Palouse, Washington	0-3-4	23	E	1×C^po^	1×D^du/po^
	0-3-10	24	1×C^du^	3×E^po^	–
			1×D^du^	3×F^po^	
	4	24	1×F^du^	3×F^po^	–
2603 Rosalia, Washington	33A	24	1×F^du^	3×E^po^	–
	33B	24	E	E	–
	2	24	E	E	–

The parental origins of the chromosomes were determined by GISH. Chromosome nomenclature of homeologous groups (A–F) followed Ownbey and McCollum [25] and Pires *et al*. [24]. Superscript letters indicate the genome origins of the chromosomes, du = *T. dubius*, po = *T. porrifolius*. Chromosomes carrying translocations are indicated by naming the chromosome according to the genomic origins of the centromeres, followed by the genome origins of the translocated segments. E-indicates chromosome set as expected from the diploid parents.

**Table 2:** Karyotype organisation of individuals of *T. miscellus* from two populations (localities of each population are indicated in column 1).

*T. miscellus* population	Plant number	Chromosome No . (2n)	Nature of karyotype imbalance		Translocations
			*T. dubius* origin	*T. pratensis* origin	
2604 Moscow, Idaho	7	24	3×F^du^	1×C^pr^	A^pr/du^
					A^du/pr^
2605 Pullman, Washington	3	24	E	E	–
	14	24	1×D^du^	3×E^pr^	–

The parental origins of the chromosomes were determined by GISH. Chromosome nomenclature of homeologous groups (A–F) followed Ownbey and McCollum [25] and Pires *et al.* [24]. Superscript letters indicate the genome origins of the chromosomes, du = *T. dubius*, pr = *T. pratensis*. Chromosomes carrying translocations are indicated as in Table 1. E-indicates parental chromosome set as expected from the diploid parent.

**Table 3:** Genomic origin of decondensed rDNA compared with previously published data from Kovarik *et al.* [22] and Matyasek *et al.* [23] using genomic–cleaved amplified polymorphic sequence (g-CAP) and reverse transcription-cleaved amplified polymorphic sequence (RT-CAP) to determine the genomic origin of rDNA sequences in the genome and the cDNA, respectively.

Population	Figure Number	Plant number	decondensed rDNA	condensed rDNA	Parental origin of genomic rDNA units determined by g-CAPS (% of *T. dubius* origin units)	Parental origin of expressed rDNA units determined by RT-CAPS, (% of *T. dubius* origin rRNA transcripts)
*T. mirus*, 2601 Pullman, Washington	Figure 2D (interphase only shown)	0-4	2×A^du^	2×A^po^	35	99
				2×D^po^		
	Figure 2A	0-7	2×A^du^	2×A^po^	28	94
				2×D^po^		
	Figure 2A	0-8	2×A^du^	2×A^po^	29	96
				2×D^po^		
*T. mirus*, 2602 Palouse, Washington		0-3-4	1×A^du^	2×A^po^	40	34
			2×D^po^	1×A^du^		
	Figure 2C	0-3-10	2×A^du^	2×A^po^	41	4
			2×D^po^			
	Figure 2C	0-4	1×A^du^	2×A^po^	47	50
			2×D^po^	1×A^du^		
*T. mirus*, 2603 Rosalia, Washington	Figure 2B	33A	2×D^p^	2×A^po^	6	97
				2×A^du^ (loci of reduced size)		
	Figure 2C	33B	2×A^du^	2×A^po^	27	97
				2×D^po^		
		2	2×A^du^	2×A^po^	27	99
				2×D^po^		
*T. miscellus*, 2604 Moscow, Idaho		7	2×A^du^	–	23	100
			2×A^pr^			
*T. miscellus*, 2605 Pullman, Washington		3	2×A^du^	–	25	54
			2×A^pr^			
		14	2×A^du^	–	nd	nd
			2×A^pr^			

Ns–FISH image not shown

Previous cytogenetic analyses revealed that both *T. mirus* and *T. miscellus* had 24 chromosomes; aneuploidy has not been previously reported in these taxa [13], [24], [25]. This chromosome number was also observed here in all but one individual of *T. mirus*, which had 23 chromosomes. It was assumed that GISH would partition the chromosome sets into 12 chromosomes of *T. dubius* origin and 12 chromosomes of either *T. pratensis* or *T. porrifolius* origin depending on the tetraploid being analysed. However, only four had an entirely balanced additive karyotype of that expected from the chromosomes of the diploid parents (Tables 1 and 2 and Figures 2 and 3). We were surprised to observe that five of the eight *T. mirus* karyotypes and two of the three *T. miscellus* karyoypes with 2*n*  = 24 chromosomes had unbalanced genomic contributions. This was manifest by some chromosomes occurring in one or three copies (monosomic and trisomic, respectively; see Tables 1 and 2). In addition, four plants, including individuals of both *T. mirus* and *T. miscellus*, carried intergenomic translocations (Figures 2A, 3A, arrows).

The *T. mirus* individuals 2603-33A and 33B are siblings from different flower heads of the same plant. Significantly, their karyotypes exhibit substantial differences; 2603-33B has an expected karyotype assuming complete additivity of parental chromosome sets, while 2603-33A is monosomic for chromosome F^du^ and trisomic for chromosome E^po^.

### Meiotic and mitotic aberrations in synthetic reconstruction of *T. mirus*

**Figure 4:**
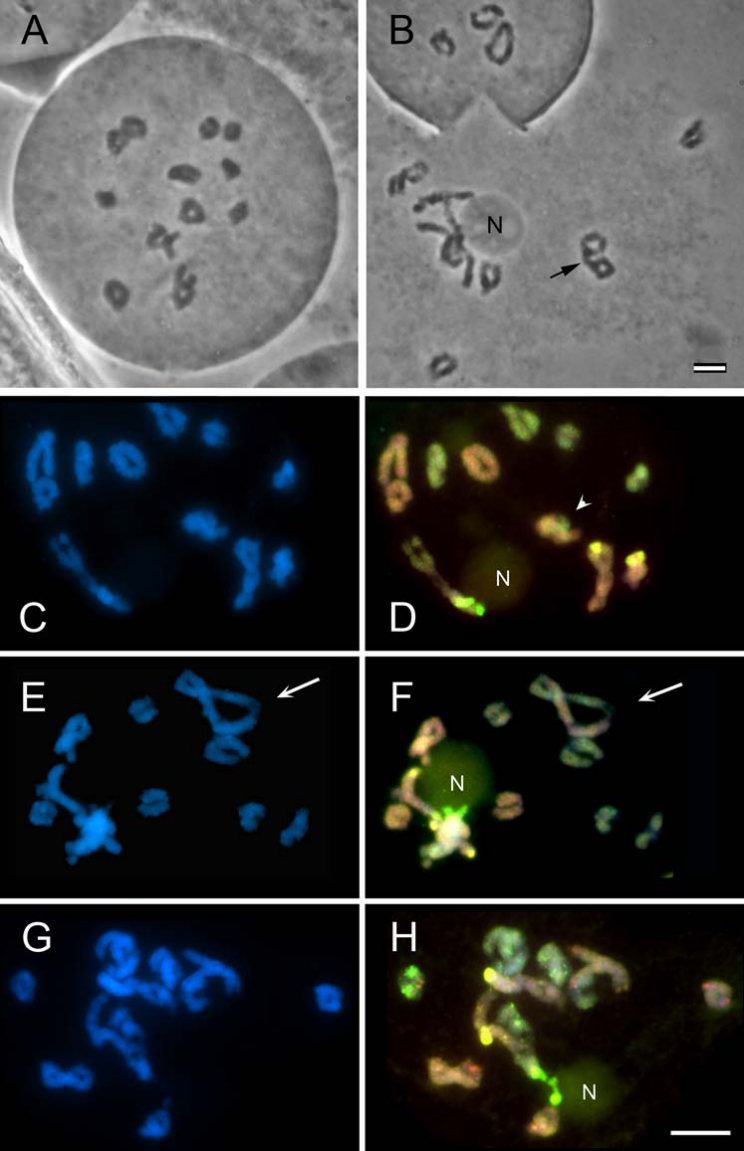
Pollen meiosis in synthetic *T. mirus.* (A,B) Feulgen staining showing in (A) 12 regular bivalents and (B) one quadrivalent (arrow) and bivalents in close association with each other and a nucleolus. (C–H). Fluorochrome colours and GISH probes are as in Figure 2. (C–D) Note chromosome A^du^ in association with the nucleolus. Two bivalents, one from the *T. dubius* genome (yellow) and one from the *T. porrifolius* genome (orange), overlapped (arrow), perhaps occurring as a multivalent. (E–F). Note the rDNA and the bivalents carrying these genes associated with the nucleolus. A quadrivalent with two chromosomes of *T. dubius* origin and two of *T. porrifolius* origin occurring in a ring (arrow). (G–H) Note two bivalents (A^du^ and D^po^) with rDNA associated with the nucleolus and the A^po^ bivalent with condensed rDNA unassociated with the nucleolus. N = nucleolus. Scale bar (C–H) is 10 µm.

Meiosis in newly formed, synthetic allotetraploids (S_0_ and S_1_) (see Figure 1) was examined to determine if meiotic aberrations occurred in early allotetraploid generations and could be a source of genomic imbalance observed in root tip metaphases. Meiotic cells of developing anthers at diplotene were analysed (Figure 4). In many instances regular bivalent formation occurred (Figure 4A), although frequently bivalents overlapped (Figure 4C, D), making it difficult to be certain if they were in fact multivalents. However, in a number of cases resolution was sufficient to determine the presence of quadrivalents (Figure 4B). Using GISH, the genomic origin of the chromosomes could be resolved, although the distinction was less clear than at metaphase. Figures 4E–F show GISH labelling of a quadrivalent with two chromosomes of *T. dubius* origin and two chromosomes of *T. porrifolius* origin physically linked via chiasma in a large, twisted ring.

**Figure 5:**
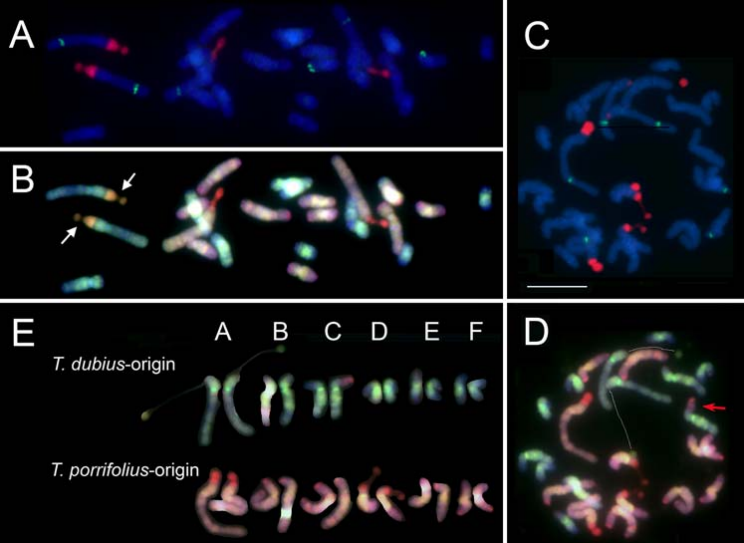
Analyses of root tip metaphases of synthetic *T. mirus* (A, B is plant 73-14; C-E is plant 134-16-3). (A, C) Metaphase (merged images) after FISH for 45S rDNA (red) and 5S rDNA (green) and counterstained with DAPI (blue). (B) and (D) Metaphases (merged images) from (A) and (C), respectively, after reprobing using GISH with labeled genomic DNA from *T. porrifolius* (pink) and *T. dubius* (green) and counterstained with DAPI. In (B) note the particularly there are only four 45S rDNA sites, the sites on A^du.^ Chromosomes are particularly large (arrows) and the site expected on chromosome D^po^ is missing. In (D) note the *T. porrifolius* translocation to a *T. dubius* chromosome (red arrow) and the satellites to both A^du^ homologues are distant from the rest of the chromosome (see connecting white lines). (E) Karyotype of (D) revealing that the *T. porrifolius* origin translocation is to chromosome C^du^ and that no *T. porrifolius* chromosomes lack chromatin. Scale bar is 10 µm.

An analysis of root tip metaphases in 12 allotetraploids of the subsequent S_1_ generation revealed one plant that was 2*n* = 23, the rest were 2*n* = 24, as expected. There were cytogenetic abnormalities in two additional plants. One of these plants (73-14) had unusually large 45S rDNA loci occurring on both copies of chromosome A^du^ (Figure 5 A, B). This plant also had an rDNA locus on chromosome A^po^ of *T. porrifolius* origin, but the expected site on chromosome D^po^ was absent. The second plant (134-16-3) had a translocation of *T. porrifolius* origin to chromosome C^du^. Since there is no missing chromosome segment of *T. porrifolius* origin, it must be assumed that this was a non-reciprocal translocation induced during the preceding meiosis (Figure 5 C, D, E).

### 45S rDNA decondensation

Previous cytogenetic analyses of diploid *Tragopogon* species revealed that *T. dubius* and *T. pratensis* carry a 45S rDNA locus on chromosome A^du^ and A^pr^, respectively, while *T. porrifolius* has two loci on chromosomes A^po^ and D^po^. To determine if these sites were inherited in the respective allotetraploid species we used FISH with digoxigenin-labelled probe pTa71 (yellow fluorescence, Figures 2 and 3). In *T. miscellus*, 45S rDNA loci occur on chromosomes A^du^ and A^pr^ (Figure 3). At metaphase both chromosomes carried secondary constrictions (Table 3). In *T. mirus*, 45S rDNA loci occur on chromosomes A^du^, A^po^, and D^po^ (Figure 2). These loci were also frequently identifiable after GISH labeling, without the use of the rDNA probe pTa71, appearing with a brighter green (to *T. dubius* origin loci) or yellow fluorescence (Figure 2). In all cells, both mitotic and meiotic, the number of 45S loci and their parental distribution were as expected, i.e. pairing and segregation of rDNA-carrying chromosomes is probably regular. This was not the case, however, for 5S rDNA-carrying chromosomes (5S probe, red fluorescence). We would expect *T. mirus* to carry 5S rDNA loci on chromosomes A^du^, A^po^ and F^po^. But here we observed one *T. mirus* individual (2602-03-10) trisomic for chromosome F^po^ and each of these chromosomes carried a single 5S rDNA locus (Figure 2). It was a surprise that the chromosomes carrying the 45S rDNA were balanced because at diplotene they were frequently in close proximity (Figure 4 E, F), probably reflecting transcriptional activity and nucleolar function. The absence of aberrant numbers of 45S rDNA-carrying chromosomes at metaphase suggests that they paired regularly at meiosis.

An analysis of 45S rDNA-carrying chromosomes in 14 diplotene nuclei of *T. mirus* revealed that chromosome A^du^ was always associated with the nucleolus (Figure 4E–H). Often this chromosome was associated with a chromosome from the *T. porrifolius* genome (when identifiable it was chromosome D^po^, Figure 4G, H). Similar results were observed at metaphase (Table 3, Figure 2), where chromosome A^du^ and sometimes chromosome D^po^ carried secondary constrictions (the exception is plant 2603-33A–below). In contrast, chromosome A^po^ was always condensed and unassociated with the nucleolus. Interestingly, in two individuals of population 2602 only one of the two homologues of chromosome A^du^ carried secondary constrictions. In plant 2603-33A, there was no secondary constriction on chromosome A^du^ and the 45S rDNA locus was unusually small. Instead, chromosome D^po^ had a secondary constriction. Similarly in two synthetic *T. mirus*, plants 134-16-3 and 73-14, there were secondary constrictions on A^du^ and A^po^, with the locus on chromosome D^po^ missing in the latter individual (Figure 5 A, C).

In natural *T. mirus* and *T. miscellus*, the presence of secondary constrictions correlated well with patterns of condensation/decondensation at interphase (Figure 2D, E); for example, chromosomes A^po^ and D^po^ have no secondary constrictions in *T. mirus* 2601-4 (not shown) and exhibit four sites of condensed rDNA chromatin (Figure 2D) while in *T. mirus* (2602-0-3-10) chromosome A^po^ is without a secondary constriction (Figure 2C) and at interphase there are two highly condensed rDNA loci (Figure 2E).

### Southern blot analysis of 5S rDNA loci

**Figure 6:**
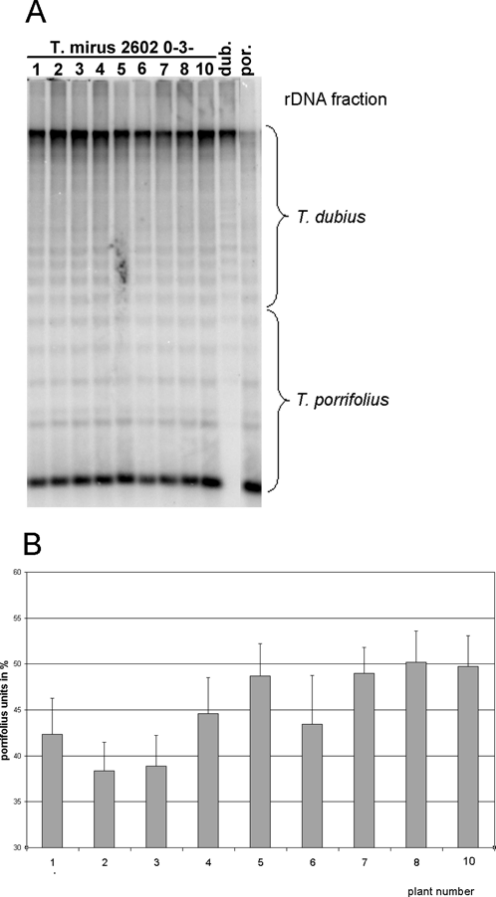
Genomic analysis of 5S rDNA repeats. (A) Southern blot hybridisation of 5S rDNA probe to *Taq*I-digested genomic DNA of the progeny of 2602-0-3 individual and diploid parental accessions. (B) Quantitative representation of the parental 5S gene families determined by a phosphorimager scanning of radioactivity signals on blots (three independent experiments).

Given the imbalance observed in the inheritance of individual chromosomes, we would expect the copy number of 5S rDNA units to vary among plants. For example, individual 2602-3-10, showing trisomy of the F^po^ chromosome, had seven 5S rDNA signals instead of the expected six (Figure 2C). On the other hand, if there was F^po^ monosomy, we might expect a reduction in gene copy numbers. Using Southern blot hybridisation, we determined the parental *T. porrifolius*/*T. dubius* 5S rRNA gene copy number ratio in the progeny of a single *T. mirus* plant, 2602-0-3. Several progeny were studied, including those analysed by GISH (plants 2602-0-3-10 and 2602-0-3-4). To ascertain the parental origin of the 5S rDNA units, we digested genomic DNA with *Taq*I restriction enzyme. This enzyme takes advantage of a restriction site present in *T. porrifolius* units that is not found in *T. dubius* units. Consequently, after digestion the *T. porrifolius* 5S rDNA was digested mostly to monomers while there was little digestion of *T. dubius* units that remained at high molecular weight (Figure 6). In *T. mirus*, the 5S rDNA probe used for Southern hybridisation revealed signals from both parental gene families. However, the distribution of signal intensities between genes of *T. porrifolius* and genes of *T. dubius* origin differed between progeny. For example, the trisomic individual 2602-0-3-10 had a *T. porrifolius*/*T. dubius* 5S rDNA unit ratio close to 1.0, while the “normal” disomic plant 2602-0-3-4 had a ratio that was 0.82. Thus, trisomy of chromosome F^po^ resulted in an approximately 10% increase in the number of 5S genes in the genome. This value is in a good theoretical agreement with the expected increase in number of 5S arrays from six to seven (per diploid cell). Three other plants (2602-0-3-5, -7 and -8) had a 5S rDNA gene ratio corresponding to a trisomic genotype, suggesting meiotic aberrations were frequent in this lineage. On the other hand, two plants (2602-0-3-2 and -3) had a decreased number of *T. porrifolius* genes compared with the expectation (plant 2602-0-3-4, with balanced chromosomal sets). Perhaps these two individuals are monosomic for F^po^.

## Discussion

### Effectiveness of GISH

Phylogenetic analyses of internal (ITS) and external transcribed spacer (ETS) sequences of nuclear 45S rDNA suggest that the diploid progenitors of both *T. mirus* and *T. miscellus* are distantly related, with *T. dubius* in one major clade of *Tragopogon* and both *T. pratensis* and the populations of *T. porrifolius* that served as parents in another major clade [26], [27]. Earlier allozyme studies also suggested that the three diploid progenitors were well differentiated genetically 16, 28. Likewise, comparisons of cDNA-AFLP genetic markers between the diploid species reveal that *T. pratensis* and *T. dubius* share only between 30-40% of markers [12], again suggesting considerable genomic divergence.

Recently, Markova et al. [29] used GISH with genomic DNA probes from diploid species of *Silene* onto metaphases of related diploids to show that labeling strength was inversely correlated to genetic distance, i.e. there was strongest labeling to the most closely related species. Given these data and an apparently large genetic distance between *Tragopogon* diploids, we might expect GISH to work effectively on the derived allopolyploids. Instead, we observed that it worked rather weakly, with much cross-hybridisation of probes, and the genomic distinction was only resolvable after electronic merging of images. Indeed previously we had reported that GISH had not worked at all in *Tragopogon* [24]. Our success here is due to improved quality of the electronics on the microscope's camera and improved facilities to electronically merge images using Openlab software, but it remains an enigma why GISH does not work more effectively in *T. mirus* and *T. miscellus*.

### rDNA inheritance and expression patterns

We expect *T. miscellus* to inherit two 45S rDNA loci (carried on chromosome A^du^ from *T. dubius* and A^pr^ from *T. pratensis*), while *T. mirus* inherits three loci (carried on chromosomes A^du^ from *T. dubius* and A^po^ and D^po^ from *T. porrifolius*). The observations of only two 45S rDNA in synthetic *T. mirus* 73-14 (Figure 5A, B) can be explained in one of two ways. First, the enlargement of the rDNA locus on chromosome A^du^ and the locus loss on D^po^ occurred through an rDNA translocation/fusion involving the loci on D^po^ and A^du^. However, given that the karyotype is balanced and the material is the S_1_ generation, this scenario seems unlikely because it would suggest the union of two gametes carrying the same abnormality, or arising via restitution of segregating chromosomes in meiosis II. The second and alternative explanation is that the rDNA locus number may reflect the situation in the *T. porrifolius* parent used for the cross, although our past analyses of *T. porrifolius* never revealed such a polymophism [24]. Unfortunately the precise *T. porrifolius* parent used in the cross is now deceased (the plants are annuals or biennials), preventing us from determining which of the competing hypotheses is correct.

Previously we showed that natural *Tragopogon* allopolyploids also do not have fixed patterns of 45S rDNA inheritance, with some individuals showing a balanced distribution of rDNA sequences originating from each of the parental diploids and some showing biased inheritance, typically with the number of rDNA units from *T. dubius* being underrepresented [22]. However, from molecular studies it was not clear whether non-Mendelian rDNA inheritance was caused by a decrease in copy number (elimination) or unit replacement (e.g. via homogenisation mechanisms). In one individual of *T. mirus*, 2603-33 (referred to here as 2603-33A), there was a considerable reduction in the number of *T. dubius* units (to ∼100 copies/diploid) from an expectation of ∼700 units (typical number for any given tetraploid population). An analysis of this individual's karyotype (Figure 2) revealed that the loss in copy number can be accounted for by a reduction in the size of the rDNA locus on chromosome A^du^. The sibling to this plant, 2603-33B, does not show this rDNA copy number deletion. Therefore, the locus size reduction in 2603-33A may have arisen from a deletion event occurring as a consequence of meiotic instability in the parent.

Despite the relatively small size (perhaps only 100 copies/diploid of the 45S rDNA unit) of the *T. dubius* 45S rDNA locus, it usually dominates rRNA expression in leaf material of *T. mirus* [23]. Even in the 2603-33A individual, with extremely reduced numbers of 45S rRNA genes of *T. dubius* origin, the locus accounts for 97% of the rRNA transcripts (see also Table 3). Nevertheless, in root-tip metaphases a secondary constriction is observed on chromosome D^po^, suggesting some transcription of the *T. porrifolius* locus occurs in that tissue, perhaps to compensate for the reduced 45S rDNA gene copy number when there is a high demand for ribosomes in metabolically active meristematic cells. In plant 2603-33B, with substantially higher numbers of *T dubius* derived 45S rRNA genes, the units of *T. dubius* origin are transcribed, and those rDNA units of *T. porrifolius* origin are silent [23]. This is reflected in the lack of a secondary constriction at the rDNA locus on chromosomes A^po^ and D^po^ in this individual (Figure 2, Table 3). The presence of secondary constrictions correlates strongly with levels of decondensation at interphase and almost certainly reflects transcriptional activity at the preceding interphase. Two individuals of *T. mirus* (2602-4 and 2602-0-3-4) show different decondensation of the two A^du^ homologues, probably reflecting genetic or epigenetic differentiation between the two homologues. In population 2605 of *T. miscellus*, all individuals investigated show only partial dominance in the expression of rDNA units of *T. dubius* origin over those of *T. pratensis* origin [23]. This is also seen in the occurrence of secondary constrictions on both rDNA-carrying chromosomes A^du^ and A^pr^ (Figure 3, Table 3).

An analysis of 45S rDNA evolution in *Nicotiana* polyploids indicates that parental loci are initially maintained in young polyploids, although the sequences within a locus may be subject to concerted evolution, and over time frames of >1 million years individual loci are lost [30]. In *Tragopogon* polyploids we do not know which of the different karyotype variants will survive selection and become fixed. Perhaps over longer evolutionary timescales interlocus homogenisation and new rDNA variants will occur and spread across all rDNA loci.

Our study shows that more than one pathway can lead to non-Mendelian inheritance of rDNA units in allotetraploids: (i) elimination or amplification of repeats within an array can occur without changes in locus number (as in the case of 45S rDNA locus in 2601-33A); (ii) a change in the number of rDNA-bearing chromosomes (and loci) without any material change in the number of genes at each locus (as with the 5S rDNA locus–Figure 6); or (iii) a combination of both, although this situation was not detected in this study.

### Meiotic irregularities

The chromosome multiplication step of polyploidy is thought to establish species isolation barriers between the newly formed polyploid and its diploid parents, whilst providing a homologous partner for each of the chromosomes. However, analyses of newly synthesised allopolyploids reveal that early-generation individuals are often infertile, or have highly reduced fertility, due to problems with meiosis including irregular pairing of homologous chromosomes [31], [32]. Selection for fecundity in synthetic polyploids is associated with generation-by-generation increased fertility [32]. Nevertheless even after many thousands of years of evolution, meiotic irregularities can still occur, as observed in *Triticum aestivum* (wheat), an allohexaploid where meiotic misdivision has been exploited in the formation of wheat aneuploid lines [33], [34].

An analysis of meiosis in the newly synthesised *Tragopogon* allotetraploids revealed the frequent occurrence of multivalents (Figure 4). Such aberrant pairing patterns may result in imbalanced chromosome contribution in subsequent generations as well as intergenomic translocations. Both abnormalities were observed in several of 12 synthetic (S_1_ generation) *T. mirus* plants analysed at metaphase. One plant was 2*n* = 23 and lacked a *T. dubius* chromosome and one plant carried a non-reciprocal translocation from a *T. porrifolius* chromosome to chromosome C^du^ (Figure 5E)

Multivalents were also observed at a low frequency in natural populations of *T. mirus* and *T. miscellus* [13] and in the few F_2_ plants resulting from diploid F_1_ hybrids of *Tragopogon* [35]. Meiotic abnormalities can cause unequal segregation of homeologous chromosomes and are a likely driver of the chromosomal imbalance between genomes observed in both *Tragopogon* allotetraploids. The two sibling plants of *T. mirus* 2603-33 had different karyoypes: one had a balanced karyotype of 12 chromosomes from each parental genome (2603-33B), while the other (2603-33A) was monosomic for chromosome F^du^ and trisomic for chromosome E^po^. Given these data, it is likely that the parent plant had a balanced karyotype, and meiotic irregularities, probably arising through multivalent formation, gave rise to the imbalanced karyotype of plant 2603-33A.

Multivalent pairing can arise either through (1) synapsis and recombination between homeologous chromosomes in meiosis I, or (2) synapsis between chromosomes carrying intergenomic translocations. Four out of 12 mitotic karyotypes in plants from natural populations had intergenomic translocations visible at mitotic metaphase following GISH (Tables 2 and 3), and additional smaller translocations, not resolvable by GISH, may also be present. Nevertheless, the quadrivalent indicated in Figure 4F (see arrow) does not show intergenomic translocations at the resolution obtained using GISH. Our analyses of plants from different populations, although sample sizes were small, showed that aberrant chromosome numbers were more prevalent for certain chromosome types. For example, chromosome A was not involved in any of the aneuploidy evetns detected, whereas chromosome F accounted for almost 40% of all subgenomic chromosome imbalances. There might be several explanations for these results. First the homology between F-type chromosomes may be larger than between other chromosomes. It is likely that the greater sequence and morphological similarities between homeologous chromosomes, the more likely there will be homeologous and multivalent pairing. In support of this, Nicolas *et al*. [36] observed in *Brassica napus* haploid hybrids that chromosomes with the highest synteny had the highest frequency of homeologous pairing. Nevertheless, in *T. mirus*, the presence of a 5S rDNA locus on F^po^ but not on F^du^ indicates some divergence between the homeologues. Secondly, all chromosomes may be equally likely to form multivalents, but aberration in copy number of some chromosomes, e.g. homeologous group A, may not be favored by selection.

The surprisingly high incidence of trisomy and monosomy in highly fertile plants with 2*n* = 24 suggests that “compensating trisomy” may be operating in natural populations of both *Tragopogon* allotetraploids. Compensating trisomy was first described by Blakeslee [37] to refer to a situation in which the loss of a normal chromosome is compensated by the presence of the two arms in new translocated associations (secondary chromosomes). That concept was extended to include the replacement of the primary chromosome by two tertiary chromosomes, or by a secondary and a tertiary chromosome [38]. Compensatory trisomy has been reported in *Datura stramonium* from progeny of a plant exposed to radium [37], [38], and from crops, including from Poales [39], [40] and tomato [41]; all were produced experimentally [42]. The putative compensatory trisomy observed here may be an example from natural plants.

The allotetraploids *T. mirus* and *T. miscellus* formed from their diploid progenitors within the last 80 years (perhaps even within the last 60 years) and given their biennial habit, the number of generations to present is likely to be less than 40 [8]. The long-term outcome of meiotic irregularities in these species is not easily predicted. On the one hand, the genomic imbalance will reduce fitness and perpetuate cycles of meiotic irregularities. This may lead to a cascade of reduced fitness, generation upon generation. Such a phenomenon was observed in synthetic *Brassica* allopolyploids maintained by single seed descent [43]. In the wild, cryptic karyotypic instability manifest by aberrant ratios of homeologous chromosomes might ultimately lead to a slow reduction in fitness and ultimately extinction. Perhaps this accounts for the loss of some local populations of both *T. mirus* and *T. miscellus* [8] and of some recently formed *Senecio* polyploid populations [6]. Nonetheless, it is noteworthy that despite meiotic irregularities in the initial synthetic S_0_ plants and subsequent S_1_ generations, pollen fertility and seed set were generally high in the synthetic *Tragopogon* polyploid lines (Tate *et al*., in prep.). In addition, selection will favour the most fertile individuals, likely to be those with the most regular chromosome pairing. Thus, if the population can expand through early bottlenecks of reduced fertility, the derived populations are likely to be more fertile with regular bivalent pairing. Certainly in well-established allotetraploids (10^4^–10^5^ myrs old), e.g. of *Nicotiana* and *Triticum*, no major imbalances in chromosome numbers or the distribution of chromosomes to subgenomes are normally observed [44], [45].

### Genomic instability

The angiosperm genome is characterised by its plasticity to genetic change, including large-scale chromosome number changes, aneuploidy and polyploidy [4]. However, it may be significant that in *Tragopogon* polyploids, all the imbalances in parental chromosome dosages between individuals occurred within a near-regular karyotype of 2*n* = 24 (one exception at 2*n* = 23). Given the frequency of plants showing genomic imbalance we might expect to find more plants with unexpected chromosome numbers. Perhaps there is selection against plants that deviate from 2*n* = 24 and against those that are nullisomic for a particular chromosome, or perhaps our sample size was too small to find a representative range of abnormalities.

GISH data revealed that in two out of three individuals of *T. miscellus* and four of nine individuals of *T. mirus,* there is an imbalance in the parental contribution of the chromosomes. However, the imbalance observed resulted from monosomy or trisomy, and no individual was nullisomic for a particular chromosome. An analysis of 10 genes in *T. miscellus* using genomic and cDNA CAPS revealed that 65% of individuals displayed losses of one of the two homeologues and a further 5% of individuals showed silencing of one of the two homeologues in leaves [12]. The missing alleles were interpreted as sequences that had been stochastically eliminated from the *T. miscellus* genome. The results here may suggest that chromosome loss or non-reciprocal translocations may contribute to the loss of alleles, although we did not find any example of a homozygous deletion for a chromosome segment. The differential expression of rDNA on the A^du^ chromosomes of two *T. mirus* individuals (Figure 2) points to epigenetic or genetic heterozygosity between the homologous chromosomes.

Large-scale genetic changes caused by parental genome imbalance will influence the inheritance of genetic markers, which will in turn influence the transcriptome, proteome and metabolome. Clearly genetic analyses of young or synthetic polyploids require in-depth cytogenetic studies to assess the contribution that chromosomal changes play in the inheritance of genetic markers. Unfortunately in recent years that work has seldom been standard practice. Such cytogenetic data are clearly needed even if chromosome counts appear regular [see also 43], since a deeper analysis of the genome and chromosome substructure can reveal substantial chromosome dosage deviation from expectation.

## Materials and Methods

### Plant material

Seeds of *Tragopogon* were collected from natural populations in Idaho (ID) and Washington (WA) (USA) (Table 1) and planted either in a greenhouse at the Department of Botany, University of Florida, or in field plots at the Institute of Biophysics, Academy of Sciences of the Czech Republic, Brno. Root tips from young, healthy, vigorously growing plants were harvested and placed in ice cold and saturated aqueous 2mM 8-hydroxyquinoline (Sigma-Aldrich Company Ltd, Poole, Dorset, UK). After 60 min incubation on ice, the roots were fixed in ethanol-acetic acid (3:1) at room temperature overnight (several washes) and stored in the same solution at −20°C until use. Developing anthers of 1 mm length or less, that contain diplotene nuclei, were excised from young buds of length 1 cm or less. Root tip and meiotic material were fixed in freshly prepared 3:1 ethanol: glacial acetic acid for two days. Root tip material was then transferred to 90% ethanol at −20°C for long-term storage.

### Making synthetic Tragopogon allotetraploids

Descriptions of the methods used to generate synthetic polyploid lines are given in detail in a separate paper reporting the formation and availability of these lines for research (Tate *et al*. in prep). Briefly, numerous repeated *T. dubius*×*T. porrifolius* crosses were made, and seeds from successful crosses germinated on moist filter paper. The chromosome number was doubled to resynthesise polyploids that closely resemble *T. mirus* by placing seedlings with fully emerged cotyledons in 0.1% or 0.25% colchicine solution overnight. After washing with water for two-three days, the seedlings were transferred to 2.5” pots with soil (and grown in the glasshouse at the University of Florida under standard conditions). Plants coded N197-3.132-1 and N197-4.98-1 were used for meiotic analyses (S_0_ generation) and root tips of selfed progeny (S_1_ generation) analysed in root tip mitosis, metaphases of plants coded 134-16-3 and 73-14 are shown.

### Feulgen staining of meiotic cells

For meiotic squashes, small developing inflorescences (1 cm or less in length) were collected from the greenhouse and fixed in 3:1 (as above, for root tips). Individual anthers were then removed and macerated in 60% glacial acetic acid, stained in aceto-orcein [46], spread under a coverslip by warming over a naked flame, and observed using a Zeiss Photomicroscope III. At least 15 meiotic cells per plant were scored.

### Preparing cell spreads for in situ hybridisation

Chromosome preparations for FISH, using either cloned probes or total genomic DNA probes (called GISH) were made using modifications of established methods [47], [48]. Briefly, root tips or developing anthers were digested in 0.3% (w/v) cellulase Onozuka R-10 (Apollo Scientific Ltd, Stockport, Cheshire, UK), 0.3% (w/v) pectolyase Y23 (MP Biomedicals, Solon, Ohio, USA) and 0.3% (w/v) drieselase (Sigma-Aldrich Company Ltd., Poole, Dorset, UK) for 28 min and transferred to 1% citrate buffer for 2 h. The meristematic cells behind the root cap were isolated in a drop of 60% acetic acid and squashed onto a glass slide. For meiotic preparation, fixed anthers were dissected and meiotic cells gently dispersed into a drop of 60% acetic acid, a coverslip applied and gently warmed. Coverslips were removed following freezing with dry ice.

### *In situ* hybridisation

Fluorescence *in situ* hybridisation followed standard protocols, Figures 2–4, Leitch *et al*. [48], Figure 5, Telgmann-Rauber *et al*. [47]. Genomic DNA from *T*. *dubius*, *T*. *pratensis*, and *T*. *porrifolius* for labelling by GISH was extracted using DNeasy Plant mini kit (Qiagen Ltd, Crawley, West Sussex, UK) following manufacturer's instructions. Genomic DNA was labelled with biotin-16 dUTP (Sigma-Aldrich Company Ltd., Poole, Dorset, UK) or digoxigenin-11-dUTP (GE Healthcare, Chalfont St Giles, Buckinghamshire, UK) using nick translation following standard protocols. The probe against 5S ribosomal DNA (rDNA) was prepared by amplifying the gene using primers described in Fulnecek et al. [49] and biotin-16-dUTP-labelling protocol as described in Leitch et al. [48]. The probe against 45S rDNA was the clone pTa71, which includes the 18- 26S rDNA subunit isolated from *Triticum aestivum*, which was labelled with digoxignenin-11-dUTP as described in Leitch et al. [48]. Briefly, slides were denatured in 70% (v/v) formamide in 2× SSC (0.3 M sodium chloride, 0.03 M sodium citrate) at 70°C for 2 min and the hybridisation mix added (4 µg.ml^−1^ labeled probes and 50% (v/v) formamide, 10% (w/v) dextran sulphate, 0.1% (w/v) sodium dodecyl sulphate in 2× SSC). *In situ* hybridisation was carried out overnight at 37°C, after which the slides were given a stringent wash (20% (v/v) formamide in 0.1× SSC at 42°C). Sites of probe hybridisation were detected using 20 µg.ml^−1^ fluorescein-conjugated anti-digoxigenin IgG (GE Healthcare, Chalfont St Giles, Buckinghamshire, UK) and 5 µg.ml^−1^ Cy3-conjugated avidin (Roche Pharmaceuticals, Lewes, East Sussex, UK) in 4× SSC containing 0.2% (v/v) Tween 20 and 5% (w/v) bovine serum albumin. Chromosomes were counterstained with 2 µg/ml DAPI (4′,6-diamidino-2-phenylindole, Sigma Aldrich Company Ltd., in 4× SSC) and stabilised in Vectashield medium (Vector Laboratories Ltd, Peterborough, UK) prior to data acquisition using either: 1) Leica DMRA2 epifluorescent microscope fitted with an Orca ER camera and Open Lab software® (Improvision, Coventry, UK) (Figures 2–4) or; 2) Olympus BX61 epiflurescent microscope using Olympus Microsuite 5 software® (Olympus America Inc, Center Valley, PA, USA) (Figure 5). The images were analysed with Adobe Photoshop® version 7 and treated for colour contrast and uniform brightness only. At least 5 mitotic or meiotic cells per plant were scored with each probe used.

### Southern hybridisation

*Taq*I restriction enzyme digestion was carried out on genomic DNA extracted from leaves using standard protocols [50] with modifications as in [51]. After fractionation in 1% agarose by gel electrophoresis, the DNA was transferred to a Hybond N+ membrane (GE Healthcare, Chalfont St Giles, Buckinghamshire, UK). The ^32^P-labelled 5S rDNA probe was from a 120-bp *Xba*I/*Eco*RI fragment of 5S rDNA cloned from *N. tabacum* [49]. Probe hybridisation was conducted under high-stringency conditions in the Church-Gilbert hybridisation buffer at 65 °C overnight. The radioactivity signals were quantified by phosphorimager scanning (Storm, GE Healthcare, UK).
